# The Susceptibility of Trypanosomatid Pathogens to PI3/mTOR Kinase Inhibitors Affords a New Opportunity for Drug Repurposing

**DOI:** 10.1371/journal.pntd.0001297

**Published:** 2011-08-23

**Authors:** Rosario Diaz-Gonzalez, F. Matthew Kuhlmann, Cristina Galan-Rodriguez, Luciana Madeira da Silva, Manuel Saldivia, Caitlin E. Karver, Ana Rodriguez, Stephen M. Beverley, Miguel Navarro, Michael P. Pollastri

**Affiliations:** 1 Instituto de Parasitología y Biomedicina “López-Neyra” Consejo Superior de Investigaciones Cientificas, Granada, Spain; 2 Department of Medicine-Division of Infectious Diseases, Washington University School of Medicine, St. Louis, Missouri, United States of America; 3 Division of Medical Parasitology, Department of Microbiology, New York University School of Medicine, New York, New York, United States of America; 4 Department of Molecular Microbiology, Washington University School of Medicine, St. Louis, Missouri, United States of America; 5 Department of Chemistry and Chemical Biology, Northeastern University, Boston, Massachusetts, United States of America; George Washington University, United States of America

## Abstract

**Background:**

Target repurposing utilizes knowledge of “druggable” targets obtained in one organism and exploits this information to pursue new potential drug targets in other organisms. Here we describe such studies to evaluate whether inhibitors targeting the kinase domain of the mammalian Target of Rapamycin (mTOR) and human phosphoinositide-3-kinases (PI3Ks) show promise against the kinetoplastid parasites *Trypanosoma brucei*, *T. cruzi*, *Leishmania major*, and *L. donovani*. The genomes of trypanosomatids encode at least 12 proteins belonging to the PI3K protein superfamily, some of which are unique to parasites. Moreover, the shared PI3Ks differ greatly in sequence from those of the human host, thereby providing opportunities for selective inhibition.

**Methodology/Principal Findings:**

We focused on 8 inhibitors targeting mTOR and/or PI3Ks selected from various stages of pre-clinical and clinical development, and tested them against *in vitro* parasite cultures and *in vivo* models of infection. Several inhibitors showed micromolar or better efficacy against these organisms in culture. One compound, NVP-BEZ235, displayed sub-nanomolar potency, efficacy against cultured parasites, and an ability to clear parasitemia in an animal model of *T. brucei rhodesiense* infection.

**Conclusions/Significance:**

These studies strongly suggest that mammalian PI3/TOR kinase inhibitors are a productive starting point for anti-trypanosomal drug discovery. Our data suggest that NVP-BEZ235, an advanced clinical candidate against solid tumors, merits further investigation as an agent for treating African sleeping sickness.

## Author Summary

In our study we describe the potency of established phosphoinositide-3-kinase (PI3K) and mammalian Target of Rapamycin (mTOR) kinase inhibitors against three trypanosomatid parasites: *Trypanosoma brucei*, *T. cruzi*, and *Leishmania sp.*, which are the causative agents for African sleeping sickness, Chagas disease, and leishmaniases, respectively. We noted that these parasites and humans express similar kinase enzymes. Since these similar human targets have been pursued by the drug industry for many years in the discovery of cellular growth and proliferation inhibitors, compounds developed as human anti-cancer agents should also have effect on inhibiting growth and proliferation of the parasites. With that in mind, we selected eight established PI3K and mTOR inhibitors for profiling against these pathogens. Among these inhibitors is an advanced clinical candidate against cancer, NVP-BEZ235, which we demonstrate to be a highly potent trypanocide in parasite cultures, and in a mouse model of *T. brucei* infection. Additionally, we describe observations of these inhibitors' effects on parasite growth and other cellular characteristics.

## Introduction

The pathogenic protozoans *Leishmania major*, *L. donovani*, *Trypanosoma brucei*, and *T. cruzi* are the causative agents for a collection of diseases that primarily affect the developing world, and are potentially lethal when untreated. Taken together, visceral and cutaneous leishmaniases, human African trypanosomiasis (HAT, or sleeping sickness) and Chagas disease affect over 22 million patients annually, causing nearly 100,000 deaths per year. Transmitted by the bite of infected insects, these diseases are treated by agents that are far from optimal in terms of safety, efficacy, and dosing methods [1], [2], [3]. Resistance to many of these therapies is emerging [4], [5], [6]. Since these diseases affect the poorest parts of the world, there is little opportunity to recover drug discovery research costs, and thus they are largely “neglected” by the biopharmaceutical industry.

The discovery of new therapeutic agents is expensive and time consuming, and various strategies have been implemented in order to mitigate costs and speed drug discovery [7]. While the pharmaceutical industry frequently begins drug discovery programs with high-throughput screening and extended medicinal chemistry research programs, this paradigm remains unaffordable for most not-for-profit endeavors to implement. Therefore, the approach of “target repurposing” is frequently employed, where molecular targets in parasites are matched with homologous human targets that have been previously pursued for drug discovery [8], [9], [10], [11]. In the best case, drugs that are selective for these human targets will have been carried into human clinical studies, strongly suggesting that the homologous parasite target is likely “druggable” [12], that is, that compounds can be designed to inhibit the target that are safe and orally bioavailable.

With an eye towards target repurposing for anti-trypanosomal drug discovery, we have identified the trypanosomal phosphoinosotide 3-kinases (PI3Ks) as a promising class of targets for pursuit. In humans, inhibition of members of the PI3K family has attracted significant interest as targets in the discovery of new anticancer and anti-inflammatory agents [13], [14], [15]. This kinase family provides critical control of cell growth and metabolism, and is comprised of three classes (I–III), as determined by structure, regulation, and substrate specificity. The Target of Rapamycin (TOR) kinase (a member of the PI3K-related kinase (PKK) subfamily) has received particular interest due to its central role in fundamental processes such as growth, cell shape and autophagy. The TOR kinases were first identified through inhibition studies with the natural product rapamycin and related compounds. This inhibition is now known to be mediated through interactions of the TOR FKBP12-rapamycin-binding (FRB) domain with the rapamycin-binding protein FKBP12 [16], [17]. More recently, inhibitors targeting the mammalian TOR (mTOR) kinase domain have been developed [18], [19], [20], [21], [22], [23]. In addition, significant effort has been employed to discover inhibitors targeting specific PI3K family members [24].

**Figure 1:**
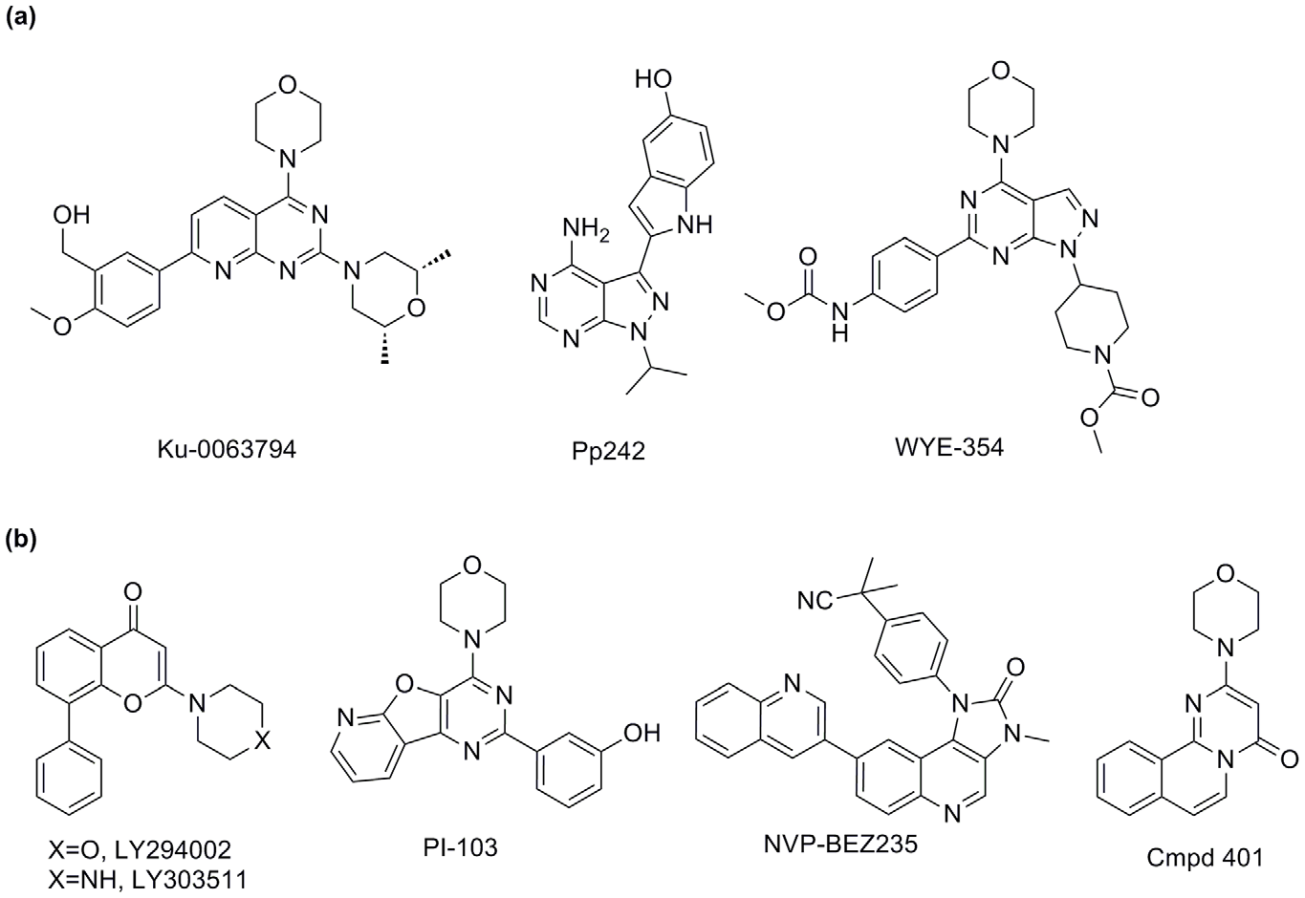
Inhibitors selected for this study. These include that are (a) selective for the mTOR kinase domain, and (b) inhibit both mTOR and human PI3Ks.

Thus far, while some agents show selectivity for mTOR or for various specific PI3Ks, selectivity is rarely absolute. Many inhibitors show broad activity against a spectrum of PI3K or TOR family members. Nonetheless, both selective mTOR and these so-called “mixed” PI3K inhibitor classes have shown promise as cancer therapeutics, suggesting that absolute specificity may not be required for therapeutic efficacy [25], [26]. Some key examples of these mTOR-selective and mixed inhibitors are shown in **Table 1** and **Figure 1**.

**Table 1:** Selectivity profile of the selected inhibitors against human enzymes.

Compound	Inhibition of Cell Growth	mTOR	PI3K				Refs.
			*p110α*	*p110β*	*p110δ*	*p110γ*	
	EC_50_ (µM)	IC_50_ (µM)	IC_50_ (µM)	IC_50_ (µM)	IC_50_ (µM)	IC_50_ (µM)	
NVP-BEZ235	0.05	<0.01	0.004	0.075	0.007	0.005	[25], [46], [58]
PI-103	0.5	0.02	0.0036	0.003		0.25	[54], [68]
LY294002		5	0.73	0.31	1.06	6.6	[69], [70]
LY303511	1						[51]
Compound 401	1	5.3	>100				[56], [71]
Pp242	0.04	0.008	2.8	2.2	0.1	1.3	[19], [20]
WYE-354	0.03	0.004	1				[21]
Ku-63794	0.03	0.01	8.9	>30	>5	>30	[22], [23]

Database mining of trypanosomatid genomes has revealed the presence of at least 12 proteins belonging to the PI3K protein superfamily (PFAM PF00454), many of which are unique to the parasites. Notably orthologous proteins are highly divergent from those of the human host. These include predicted kinases related to the eukaryotic class I and II PI3Ks, PI4Ks, and PIKKs including TOR, ATM and ATR ([27], [28], and data not shown). Where tested, PI3Ks appear to be essential for viability and/or virulence in trypanosomatids. Two PIK subfamily members have been examined in *T. brucei*. The trypanosome Class III PI3K TbVps34 has an essential function in membrane trafficking and in Golgi segregation during cell division [29]. These authors suggested that, similar to yeast, *T. brucei* possesses only one genuine PI3K. TbPI4Kβ is also an essential protein in *T. brucei*, required for maintenance of Golgi structure, protein trafficking, and cytokinesis [29]. Trypanosomatids possess four distinct genes belonging to the TOR family, in contrast to mammals, which possess a single mTOR protein [30], . TORs act in concert with other proteins in complexes referred to as TORCs, which have different protein subunit compositions, and cellular functions [34].

In *T. brucei*, the two conserved signaling complexes, TORC1 and TORC2, whose functions appear analogous to that described in mammalian or yeast TORCs, mediate the essential functions of *TOR1* and *TOR2* for cell growth [33], [35]. While TbTORC1 regulates protein synthesis, cell cycle progression and autophagy, TbTORC2 plays a key role in maintaining the polarization of the actin cytoskeleton, which is required for the proper functioning of endocytic processes, cell division, and cytokinesis [30], [36]. Correspondingly, *TOR1* and *TOR2* are essential genes in *Leishmania major* [31]. Recent work has characterized a third TOR protein, TOR3, in *Leishmania major* and *T. brucei*, that is implicated in the formation of acidocalcisomes and participation in stress response [31], [32]. A fourth TOR in *T. brucei* and *Leishmania* (TOR4) lacks the FRB domain responsible for binding rapamycin-binding proteins, yet possesses all other characteristic domains of TOR kinases [30], [31].

The essentiality of several PIKs and *TOR1* and *TOR2* and the requirement for *TOR3* for virulence in both trypanosomes and *Leishmania* provide genetic validation of these essential kinases as potential drug targets. Since rapamycin analogs are relatively modest inhibitors of trypanosomatid TORs and/or parasite growth [30], [31], [37] and difficult to synthesize, we focused in this work on kinase domain inhibitors under development. As these kinase domain inhibitors are generally more drug-like, soluble, and synthetically accessible than rapamycin analogs, we anticipate these properties could facilitate future optimization efforts.

## Materials and Methods

### Ethics statement

The animal experimental protocol (2010102/1) used for African trypanosome studies was reviewed and approved by the Ethical Committee IPBLN-CSIC of the Spanish Council of Scientific Research (CSIC). For *T. cruzi*, animal studies were approved by the Institutional Animal Care and Use Committee of New York University School of Medicine (protocol #81213), which is fully accredited by the Association For Assessment and Accreditation Of Laboratory Animal Care International (AAALAC). For *L. major*, animal studies were approved by the Animal Studies Committee at Washington University (protocol #20090086) in accordance with the Office of Laboratory Animal Welfare's guidelines and AAALAC.

### Inhibitor compounds

Inhibitor compounds were received from commercial vendors and used as received. PI-130, NVP-BEZ235, Ku-0063794, Pp242, and WYE-354 were obtained from Chemdea, Inc. (Ridgewood, NJ). LY294002, LY303511, and Compound 401 were obtained from Tocris Biosciences (Ellisville, MO).

### Potency assessment against *T. brucei*

Assays were performed using the strain of *T. brucei brucei* Lister 427 adapted to the laboratory, and the human-infective strain *T. b. rhodesiense* (EATRO3 ETat1.2 TREU164 [38]). Both strains were grown and tested as bloodstream forms. To establish the EC_50_, cultures of *Trypanosoma brucei* and *T. b. rhodesiense* were treated with two-fold increasing concentrations of compounds (with similar DMSO increasing concentration as control). We also utilized *T. b. gambiense* strain Eliane MHOM/CI/52/ITMAP 2188, and another *T. b. brucei*, strain 927/4 GUTat10.1 [38]. Cell populations were measured at 72 hours with an Infinite F200 microplate reader (Tecan Austria GmbH, Austria); the determination of cell viability was carried out by the established colorimetric technique AlamarBlue® with modifications, a 96-well plate format spectrophotometric assay which measures the ability of living cells to reduce resazurin [39], [40]. Data obtained with *T. b. brucei* Lister 427 were confirmed by manual counting in a Neubauer chamber for a direct microscopic examination to rule out multinucleated phenotypes that could mask the colorimetric assays, as well as the subtraction of solvent background to dismiss a potential solvent-derived fluorescence. Pentamidine was used as drug control for potency comparison, and *T. b. brucei* Lister 927 strain was included in our experiments to evaluate the adaptation to medium for the different strains as a variable condition.

### Analysis of morphological and cell cycle alterations from compound treatment in *T. brucei*

Flow cytometry was used to assess cell size and DNA content, to reveal a G1 or G2 arrest and multinucleated cells. Briefly, bloodstream cells of *T. brucei brucei* Lister 427 strain in early log phase culture were treated with high dose (1 µM for PI-103, 2 µM for WYE-354 and Pp242 and 100 nM for NVP-BEZ235) of compounds for 16 hours, when the cells were pelleted and washed to remove all traces of drug. After permeabilization with 1 µL saponin (0.5 mg/mL final concentration), the culture was RNAse treated for 30 minutes (10 µg/mL final concentration) and stained with 20 µg/mL propidium iodide immediately before its acquisition in a FACscan cytometer. Cells incubated with equivalent concentration of drug solvent (DMSO) were included in each experiment as control population.

### Potency assessment against *T. cruzi*

*T. cruzi* trypomastigotes from the Tulahuen strain stably expressing the β-galactosidase gene [41] were obtained from the supernatant of infected cultures of LLC-MK2 cells harvested between days 5 and 7. To remove amastigotes, trypomastigotes were allowed to swim out of the pellet of samples that had been centrifuged for 7 min at 2500 rpm.

For measurement of intracellular replication, 5×10^4^ NIH/3T3 cells and 5×10^4^ trypomastigotes per well were seeded in 96-well plates in DMEM supplemented with 2% FBS and Pen-Strep-Glut. DMEM did not contain phenol red to avoid interference with the assay absorbance readings at 590 nM. After 3 hours, compounds were added to a final volume of 200 µL/well at the indicated concentrations and mixed by pipetting. A 4 µM Amphotericin B solution (Sigma-Aldrich) was used as positive control. After 4 days of incubation at 37°C 5% CO_2_, 50 µL of PBS containing 0.5% of NP40 and 100 µM chlorophenol red-β-D-galactoside (CPRG) (Fluka) were added to each well. Plates were incubated at 37°C for 4 hours and absorbance was read at 590 nm.

For evaluation of extracellular survival, free trypomastigotes were rinsed once and placed in 96-well plates at 100,000/well with the compounds in a final volume of 200 µL of DMEM (without phenol red) supplemented with 2% FBS, Pen-Strep-Glut and 100 µM CPRG. Plates were incubated for 24 h at 37°C and absorbance was read at 590 nm.

### Potency Assessment against *Leishmania*

*Leishmania major* strain FV1 (MHOM/IL/80/Friedlin) was grown in M199 media [42]. *Leishmania donovani* strain LdBob (MHOM/SD/62/1S-CL2D) were grown in modified M199 media as promastigotes (26°C) [43]. Amastigote specific media (37°C) was used for growth and differentiation of amastigotes [43]. *L. donovani* axenic amastigotes were passed once following differentiation prior to use. Cells were enumerated using a Coulter Counter (BD Biosciences); as amastigotes tend to grow in clumps, *L. donovani* axenic amastigotes were passed gently through a blunt 27-gauge needle prior to counting. For determination of EC_50_ values, log phase cells were inoculated at concentration of 10^5^/ml into appropriate media with compounds as indicated, and counted when the controls lacking drug had reached late logarithmic phase. The EC_50_ is defined as the concentration of drug inhibiting 50% of control growth, and was calculated by linear regression analysis using SigmaPlot 2000.

### Cell size and DNA content analysis in *Leishmania*

*L. major* log phase promastigotes were inoculated at a concentration of 10^6^ cells/ml into media with compounds as indicated, and incubated overnight with varying drug concentrations to assess cell size and DNA content. For cell size, forward scatter of live promastigotes was measured by a FACS flow cytometer (Becton Dickinson), utilizing dye exclusion with 5 µg/ml propidium iodide (PI) to gate for live cells. DNA content was determined by flow cytometry using fixed and permeabilized *L. major* stained with PI as previously described [44], [45], but reducing the incubation time with PI and RNase A from 1 hour to 30 minutes. Histogram analysis was performed using CellQuest 3.1 software (BD Bioscience).

### Drug dosage for *in vivo* experiments

The targeted dosage of inhibitors was determined based on the pharmacokinetic studies disclosed by Maira, *et al.* [46]. Our goal was to test NVP-BEZ235 in the animal models at the highest dose achievable without inducing toxicity. For *L. major*, 12.5 mg/kg orally was the highest tolerable dose while 30 mg/kg intraperitoneally (ip) was used for the *T. cruzi* infections. A lower dose was initially used in *T. brucei*, 5 or 10 mg/kg intraperitoneally.

### *In vivo* drug evaluation in *T. brucei*

Female Balb/C mice (Jackson Laboratories, Bar Harbor, ME) were infected with 10^4^ cells of an early log phase culture of *T. b. rhodesiense* EATRO3; 72 hours after infection the mice were arbitrarily separated into three independent groups, daily treated with 5 or 10 mg/kg NVP-BEZ235, 20 mg/kg pentamidine, or DMSO, via intraperitoneal injection for four days. The parasitemia was checked at days 3, 5, 7, 11 and 14 post-infection in alive mice: in those cases the parasitemia was too low to detect by Neubauer chamber count, the extracted blood was incubated in a 24-well plate with HMI-9 medium supplemented with 20% SBFi at 37°C with 5% CO_2_, and positive wells were confirmed by direct visualization of parasites. Humanitarian sacrifice was executed, according to Ethic Commission of Animal Welfare directions, and necropsies were done in order to identify any physical side effect related to administration.

### *In vivo* drug evaluation in *T. cruzi*

Balb/c mice were inoculated intraperitoneally with 10^5^ trypomastigotes from *T. cruzi* Y strain expressing firefly luciferase (kindly provided by Dr. Barbara Burleigh, Harvard University). On day 7 post infection, mice were anesthetized with ketamine/xylazine and injected with 3 mg of D-Luciferin Potassium Salt (Gold Biotechnology) at 20 mg/ml in PBS and imaged in the IVIS Lumina II (Caliper Life Sciences). On day 8, groups of five mice were injected intraperitoneally with either 30 mg/kg of NVP-BEZ235 in DMSO or only DMSO, as control. Mice were treated for 5 days and imaged again on day 13. Data is expressed as the ratio between luciferase units in day 13 versus day 7 to determine the progression of infection with and without drug treatment.

### *In vivo* drug evaluation in *L. major*

Mice were infected with luciferase expressing *L. major* (LmFV1LucTK-1) and analyzed by bioluminescent imaging as described [47]. Balb/c mice were infected with 10^5^ *L. major* metacyclic stage parasites purified by gradient centrifugation [48]. Luminescence was measured using an IVIS 100 instrument and analyzed with Living Image software version 2.60. NVP-BEZ235 was resuspended in DMSO and applied at 12.5 mg/kg/day by oral gavage for 10 days, with treatment starting day 17 post infection. At this dose the mice showed significant weight loss, suggesting that this dosage was the highest practicable, as dosing intraperitoneally at 25 mg/kg/day was lethal.

### List of accession numbers

The following trypanosomatid enzymes are discussed in the text: LmjF36.6320 (LmjTOR1), LmjF34.4530 (LmjTOR2), LmjF34.3940 (LmjTOR3), LmjF20.1120 (LmjTOR4), Tb927.8.6210 (TbVps34), Tb927.4.1140 (TbPI4K beta), Tb927.3.4020 (TbPI4K alpha), Tb927.10.8420 (TbTOR1), Tb927.4.420 (TbTOR2), Tb927.4.800 (TbTOR3) Tb927.1.1930 (TbTOR4), Tb11.01.6300 (TbATR), Tb927.2.2260 (TbATM).

## Results

### Compound selection

We selected eight commercially-available compounds (**Figure 1**, **Table 1**) to profile for activity against *Trypanosoma brucei*, *T. cruzi* and two species of *Leishmania*, cutaneous *L. major* and visceral *L. donovani*. In order to identify potential inhibitors of trypanosome TORs or PI3Ks, we selected a range of compounds with varied potencies and selectivities against mTOR/PI3K. In mammalian cells, compounds Ku-0063794 [22], [23], Pp242 [19], and WYE-354 [49] inhibit the kinase domain of mTOR selectively with low nanomolar IC_50_ values. LY294002 is a mixed inhibitor targeting both mTOR/PI3K [50], and many analogs have been made (including LY303511, which inhibits mTOR-dependent and independent pathways, but does not inhibit PI3Ks [51], [52]). PI-103 inhibits PI3Ks with high potency and mTOR with a reported 20 nM IC_50_ [53], [54], [55]. Compound 401, a compound structurally related to LY303511, inhibits mTOR and cellular growth at low micromolar concentrations [56], while NVP-BEZ235 inhibits both PI3Ks and mTOR with sub-nanomolar IC_50_ values [57], [58].

### *In vitro* testing of inhibitors

We first tested these compounds against parasites grown *in vitro*. For *T. brucei* and *Leishmania donovani*, it is possible to cultivate free parasites *in vitro* as the infective stage forms: bloodstream form (BSF) for *T. brucei*, and axenic amastigotes for *L. donovani*. Compounds were also tested against *L. major* promastigotes (the stage carried normally by the insect vector). To study infective forms of *T. cruzi*, compounds were added simultaneously with trypomastigotes to 3T3 fibroblast host cells and incubated for four days. This protocol thus monitors all steps of the *T. cruzi* infective cycle (entry, differentiation and replication as amastigotes) as well as potential effects mediated through host cell PI3Ks. The results of the *in vitro* assessment of this inhibitor collection are shown in **Table 2**.

**Table 2:** Summary of potency data of mTOR/PI3K inhibitors against trypanosomatid cultures.

	*Leishmania sp.*			*Trypanosoma sp.*		
Compound	*L. major* a	*L. donovani* a	*L. donovani* b	*T. cruzi* c	*T. b. brucei* d	*T. b. rhodesiense* d
PI-103	0.32±0.16 *	1.05±0.28	0.62±0.41	>25	0.214±0.036	0.105±0.01
NVP-BEZ235	0.11±0.05	0.14±0.08	0.07±0.04	0.12	<10 nM	0.73±0.06 nM
LY294002	>25	-	>5	-	>2	-
LY303511	>25	-	>5	>10	>2	-
Compd 401	>25	-	>5	>10	>10	-
Pp242	2.4±0.8 #	0.42±0.03	0.50±0.09	>10	0.48±0.1	0.166±0.015
WYE-354	4.1±0.3*	5.95±0.84	6.10±1.73	>10	0.58±0.26	0.78±0.08
Ku-63794	>25	-	>5	>10	0.89±0.14	-

a *promastigotes, average of three replicates;*

b *axenic amastigotes, average of three replicates;*

c *trypomastigotes, average of three replicates, within ±10.2%;*

d *bloodstream form, average of three replicates.*

*: *p<0.05 for L. major vs. L. donovani promastigotes;*

# *p<0.05 for L. major promastigotes vs. L. donovani promastigotes or amastigotes.*

Effective concentration (EC_50_) values are shown in micromolar concentrations except as noted.

**Figure 2:**
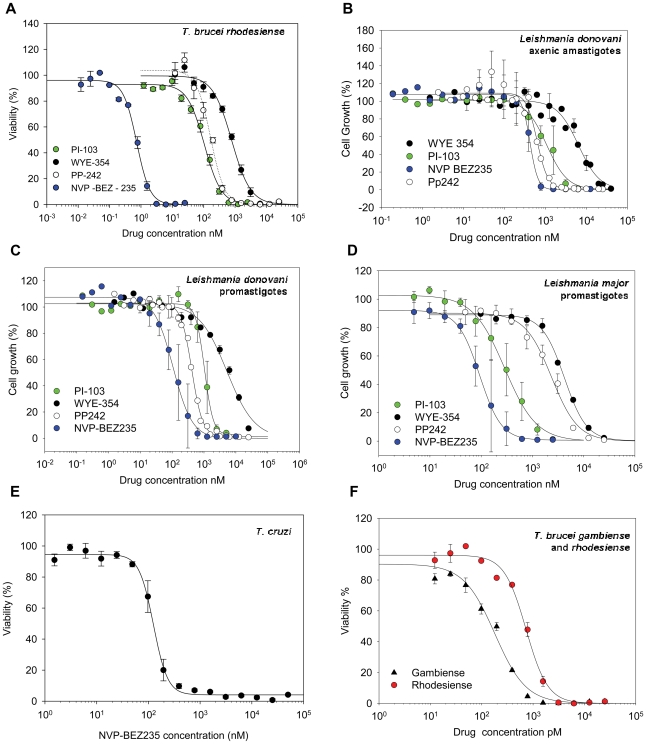
Dose response curves of the most active inhibitors. PI-103, WYE-354, Pp242 and NVP-BEZ235 against (A) *T. brucei rhodesiense*, (B) *Leishmania donovani* axenic amastigotes, (C) *Leishmania donovani* promastigotes, (D) *Leishmania major* promastigotes; (E), NVP-BEZ235 against *T. cruzi*, and (F) *T. brucei rhodesiense* and *gambiense*.

The most potent compound against all the species tested was NVP-BEZ235, showing nanomolar potency against BSF *T. brucei brucei* (Lister 427) and sub-nanomolar activity (730 pM) against the human-infective EATRO3 strain of *T. b. rhodesiense* (**Figure 2A, F**). Interestingly, the BSF *T. brucei gambiense*, ELIANE strain [38] was even more sensitive, with an EC_50_ of 179 pM. PI-103 showed good activity against *T. b. brucei* and *T. b. rhodesiense* cultures (200 and 100 nM, respectively). The other inhibitors showed micromolar activity against *T. brucei brucei*, and, as observed with NVP-BEZ235, these inhibitors are approximately ten-fold more potent against *T. b. rhodesiense*. The variation in potency of NVP-BEZ235 across different strains of *T. brucei*, (including the *T. brucei brucei* 927/4 GUTat10.1 strain) is comparable to that seen in similar studies of pentamidine, an established drug (**Table 3**).

**Table 3:** Summary of potency data of NVP-BEZ235 against *T. brucei brucei*, *T. b. rhodesiense*, and *T. b. gambiense*, compared to the known drug pentamidine.

	EC_50_ (nM)	
	NVP-BEZ235	Pentamidine
*T. b. brucei* 427	16.3±4.7	4.2±0.2
*T. b. brucei* 927	1.7±0.5	30.3±10.4
*T. b. rhodesiense* EATRO3	0.73±0.06	3.7±0.4
*T. b. gambiense* ELIANE	0.18±0.2	2.5±0.5

**Figure 3:**
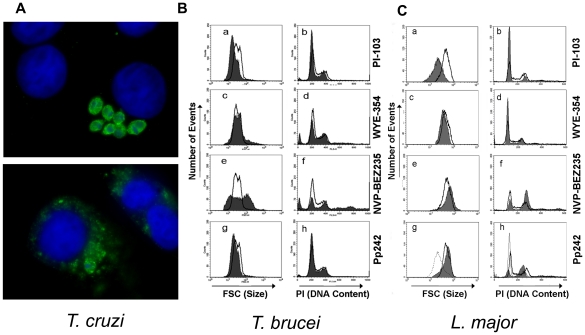
Phenotypic observations of parasites upon dosage with NVP-BEZ235. (A) NIH-3T3 host cells were incubated with *T. cruzi* trypomastigotes for 2 h before addition of NVP-BEZ235 (350 nM). Cells were incubated for 4 days during which time the parasites differentiate and replicate as amastigotes. At that time cells were fixed and stained with an anti-*T. cruzi* antiserum (green) and DAPI to visualize DNA (blue). The upper panel shows control cells with intact amastigotes, and the lower panel shows debris of parasite proteins throughout the host cell cytoplasm. (B,C) Fluorescence-activated cell sorter (FACS) analysis of cell size (Forward Scatter, FSC) and DNA content after drug treatment. (B) Bloodstream form culture of *T. b. brucei* was subjected to different drugs, indicated to the side, and analyzed by FACS for cell size and DNA content stained by propidium iodide. Cell cultures were incubated during 16 h with PI-103 (1 µM), WYE-354 (2 µM), Pp242 (2 µM) and NVP-BEZ235 (0.1 µM), represented with dark lines, and with DMSO as control population, represented as shaded area. (C) Treatment of *Leishmania major* promastigotes. Dark lines are WT parasites examined when in logarithmic growth phase; shaded areas are parasites grown in the presence of the indicated concentration of drug. Cell size (FSC) and DNA content (PI staining) were determined as indicated in Panel B and/or as described in the Methods. The subpanels show data for PI-103 (4 µM, ∼EC_90_); WYE-354 (25 µM, ∼EC_60_); NVP-BEZ235 (0.5 µM, EC_90_) and Pp242 (dashed lines 12.5 µM/∼EC_90_, shaded area 25 µM/∼EC_90_).

In infections of host cell fibroblasts by infective trypomastigotes, *T. cruzi* was refractory to all the inhibitors tested, except for NVP-BEZ235 (EC_50_ = 120 nM, **Figure 2E**). For this compound amastigotes lysis within host cells was observed after three days when the drug was dosed at 350 nM (∼3× the EC_50_; **Figure 3A**). In contrast, NVP-BEZ235 showed little activity (EC_50_ >50 µM) against free trypomastigotes, which do not replicate outside of host cells. This suggests that NVP-BEZ235 could act specifically against the amastigotes stage, or by activation of host cell responses.

For *Leishmania*, NVP-BEZ235 and PI-103 showed submicromolar inhibition across both species and stages (70–140 or 320–1050 nM respectively), while Pp242 and WYE-354 showed modest activity (0.4–2.4 µM or 4–6 µM respectively, **Figure 2B–D**). The remaining four inhibitors (LY294002, LY303511, Compound 401 and Ku-63794) were inactive against *L. major* promastigotes and *L. donovani* axenic amastigotes at the highest concentration tested and were not tested against *L. donovani* promastigotes. While some compounds showed statistically significant differences amongst the *Leishmania* strains/species, the differences were modest and not studied further.

### Phenotypic effects elicited by PI3K inhibitor treatments

We examined effects of several of the strongest inhibitors on cell size, shape and/or DNA content, since mTOR and PI3K inhibitors affect the size of both mammalian and *T. brucei* cells and induce characteristic growth phase arrests [30], [59]. While these studies cannot determine unambiguously which of the numerous members of the trypanosomatid PI3K family may be targeted, they provide a preliminary sense of the mode of action. Indeed, the molecular target in each parasite may actually be different.

Sixteen hour treatment of BSF *T. brucei brucei* with drug at an effective concentration (described below) produced two different types of effects on cellular DNA content (**Figure 3B**). Two drugs, (PI-103 and Pp242, tested at 1 µM and 2 µM, respectively) induced G1 arrest, an effect maintained even at low concentrations of Pp242 (200 nM, data not shown). While the inhibitor PI-103 showed a clearly defined profile in cell cycle progression, NVP-BEZ235 produced a combination of effects on the cell cycle progression at 0.1 µM, including the appearance of zoids (anucleated cells) [60] and multinucleated cells. This relatively high dose of NVP-BEZ235 (10× the EC_50_) produced a reduction of G1 and G2 cells. Finally, treatment of *T. b. brucei* cells with WYE-354 resulted in no significant variations in cell cycle, with a small but noticeable reduction in cell size.

We examined the effect on cell size and DNA content for the four compounds that displayed activity against *L. major* promastigotes, using drug concentrations (EC_60_-EC_90_) that were strongly inhibitory, but without inducing complete growth arrest or cellular toxicity (as evidenced by PI exclusion tests, not shown). For all concentrations tested, both PI-103 and WYE-354 treatment induced a G1 arrest and a decrease in cell size (**Figure 3C**) as seen for mTORC1 inhibitors in mammalian cells. In contrast, NVP-BEZ235 treatment induced G2 growth arrest and increased promastigote size in a manner similar to the cell phenotype observed in mammalian cells exposed to mTORC2 inhibitors. Microscopy data suggests that the G2 arrest was actually due to altered cytokinesis, as evidenced by the abundance of individual cells that contain 2 nuclei and kinetoplasts (data not shown), again consistent with known effects of mTORC2 inhibition in mammalian cells. Though PI-103, WYE-354 and NVP-BEZ235 generated single phenotypes, Pp242 generated two different phenotypes depending on the drug concentration. At lower concentrations, Pp242 induced a decrease in cell size and a G1 arrest, while at higher concentrations a G2 arrest and increase in cell size was observed (**Figure 3C**). This suggests the likelihood of inhibition of multiple targets with various affinities within the parasite.

### *In vivo* tests

We chose the most active inhibitor, NVP-BEZ235, for testing in appropriate animal models of *T. brucei rhodesiense*, *T. cruzi*, and *L. major* infection. Using the highest tolerable doses appropriate for each infection model, no efficacy was observed against either *T. cruzi* (30 mg/kg, 5 days, intraperitoneal) or *L. major* (12.5 mg/kg/day, 10 days, oral gavage) (data not shown). Weight loss was observed in drug-treated mice infected with *L. major* and higher drug doses were lethal.

**Figure 4:**
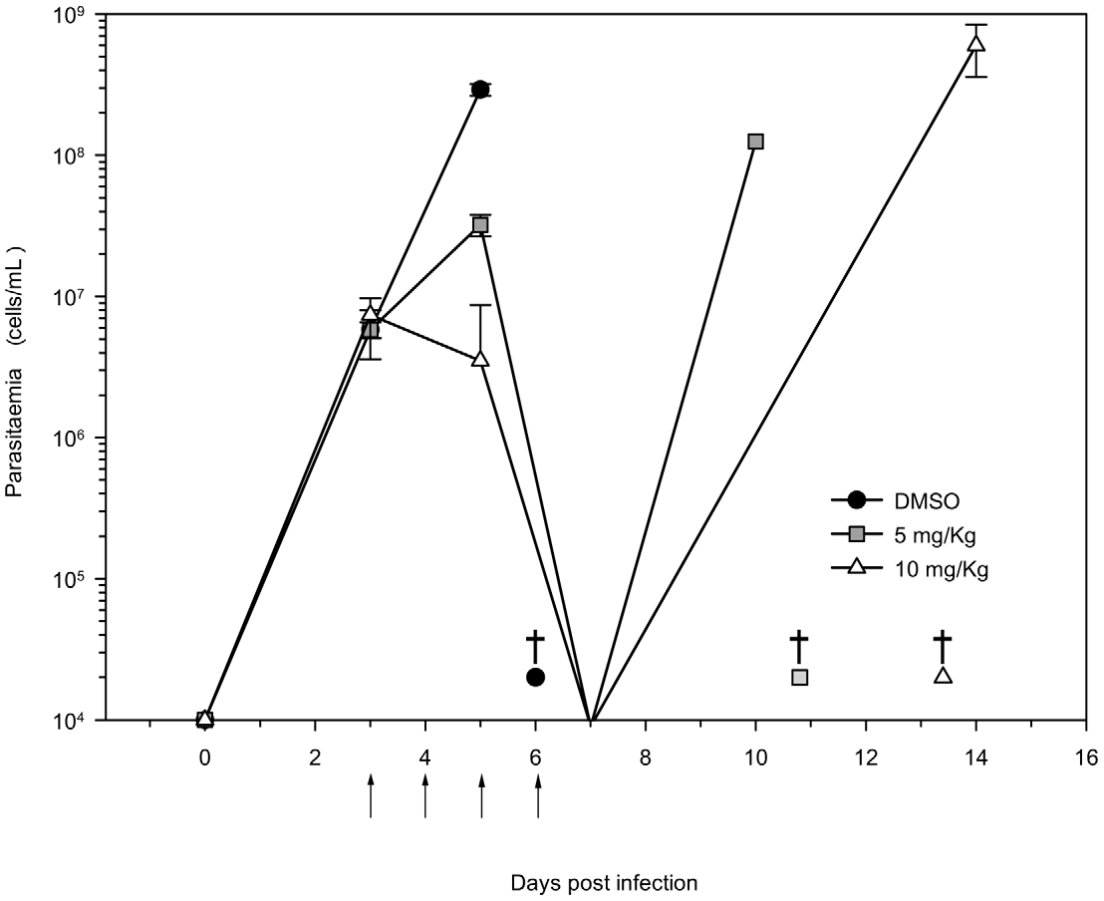
Trypanocidal activity of NVP-BEZ235 in an acute mouse infection model. Three independent groups (n = 5 per dose group) were infected with *T. b. rhodesiense*, and treated with DMSO, 5 mg/kg or 10 mg/kg of NVP-BEZ235, intraperitoneally, once a day. The arrow indicates the drug dosing schedule. The mean parasitemia for each group is represented for each day up to the death of all mice in a group. The mean survival day (MSD) is labeled in the graphic with daggers.

In contrast, a marked decrease in parasitemia was observed by intraperitoneal dosage (5 or 10 mg/kg) of NVP-BEZ235 in *T. brucei rhodesiense* infected mice. Drug was administered once per day, for four days. A dramatic decrease in parasitemia was observed within two days, below the detection limit of 10^4^ parasites/mL. All mice in the untreated group died the 6th day post-inoculation, while the mean survival day (MSD) for animals treated with 5 mg/kg of NVP-BEZ235 was extended to 10.8 (±2.4) days. The MSD of mice treated with 10 mg/kg increased to 13.4 (±3.3) days, doubling the survival of the control group (**Figure 4**). In comparison, parasitemia was below detectable limits after two days of treatment with pentamidine (20 mg/kg, ip [61]), and parasite counts remained below these limits for 30 days past dosing (data not shown).

## Discussion

The intent of these experiments was to identify promising small molecules that could represent a starting point for further medicinal chemistry optimization and a better understanding of molecular pharmacology. Noting the functional and structural homology between TOR and PI3Ks in humans and trypanosomatids and encouraged by the remarkable growth inhibitory phenotype resulting from depletion of TOR in *T. brucei* [30] and *L. major* [31], we identified and procured eight established inhibitors for assessment against parasite cultures. Several of these inhibitors inhibited parasite growth in all species/strains tested, and one, NVP-BEZ235, reduced parasitemia in an animal model of *T. brucei rhodesiense* infection, significantly extending survival. NVP-BEZ235 showed very potent inhibition of *T. brucei* growth, with a phenotype similar to that seen previously in genetic studies of *TOR* [30] and Tb*Vps34* [29]. A modest treatment regime (10 mg/kg, for four days) was able to eliminate 80% of the parasites in *T. b. rhodesiense* infections. The *in vitro* potency (179 pM EC_50_) observed against *T. b. gambiense* was exceedingly high, at a level rarely seen against these protozoan parasites. Given these encouraging results, we believe that additional studies of NVP-BEZ235 are warranted in the future, with the goal to determine whether an NVP-BEZ235 dosing regimen may be formulated able to achieve therapeutically useful effects.

The reason for the difference in potency between *T. b. rhodesiense* and *gambiense*, and between *T. b. brucei* Lister 427 and 927, is not known at this time. (**Table 3**). *T. b. gambiense*, with a lower generation rate and poorer adaptation to culture than *T. b. rhodesiense*, may be more affected by an antiproliferative drug as NVP-BEZ235. The potency of this compound and the complex cell cycle phenotype observed suggest that the compound likely has a number of molecular targets in *T. brucei*, perhaps affecting other essential cellular functions besides cell proliferation.

*T. cruzi* was relatively insensitive to the inhibitors compared to *T. brucei*. This may arise from the fact that *T. cruzi* trypomastigotes, the form of the parasite that proliferates in the human, only replicates in the intracellular environment. As a consequence, compounds need to cross the plasma membrane of the host cell to have access to *T. cruzi*, while *T. brucei* is directly accessible to the drugs in the bloodstream. When NVP-BEZ235 was tested against free, non-replicating *T. cruzi* trypomastigotes, it was inactive, while it induced lysis of intracellular *T. cruzi* amastigotes. This raises the possibility that this compound acts either specifically against the amastigote stage, or through effects on host cell PI3Ks, or some combination. The involvement of host cell PI3K and mTOR pathways in immune evasion has been recently reported for *L. donovani* [62].

*Leishmania* showed a range of sensitivities to the panel of inhibitors, with the most potent compounds active at sub-micromolar concentrations. This may be compared to the efficacy of current front line anti-leishmanial agents, whose potencies when measured by methods similar to those described here range from 30 nM for amphotericin B to 15 µM for antimonial based compounds [63].

The EC_50_ values for the compounds tested here were similar for *L. major* promastigotes, *L. donovani* promastigotes and *L. donovani* axenic amastigotes, suggesting that preliminary screening against a single form could be sufficient in the future. However, despite its potency against *L. donovani* axenic amastigotes, high doses of NVP-BEZ235 against *L. major* infections of mice showed no therapeutic effect (data not shown). As discussed above for *T. cruzi*, the lack of efficacy in the animal model of *L. major* could reflect a similar need for the drug to traffic to the phagolysosomal compartment where *Leishmania* reside. The 12.5 mg/kg oral dosing regimen tested here resulted in severe weight loss (not shown) suggesting attempts to treat with higher doses of NVP-BEZ235 would result in significant toxicity.

Current data suggest there are at least 12 members of the PI3K protein superfamily, for which the phenotypic effects of inhibition or genetic deletion in *T. brucei* or *Leishmania* are known for only five. Thus, it is difficult to assess from our studies what the likely cellular target may be. However, with an eye towards initial identification of specific targets potentially involved in the activity of NVP-BEZ235, we note a similar cell cycle phenotype that Hall *et al.* reported upon RNAi knockdown of TbVsp34 [29], including the appearance of zoids, multinucleated cells and reduction of G1 and G2 (**Figure 3B**). Barquilla *et al.* also showed the same phenotype after RNAi of *TOR2* [36].

In trypanosomes and mammals, TORC1 inhibition is known to result in G1 arrest and decreased cell size, while TORC2 results in G2 arrest and increased cell size [30], [34], [64], [65], [66], [67]. In both *T. brucei* and *Leishmania* PI-103 resulted in G1 arrest and cell size reduction, while NVP-BEZ235 resulted in aberrant cell cycle and multiple cell sizes (**Figure 3**). WYE-354 also resulted in G1 arrest/cell size decrease in *Leishmania*. In contrast, while Pp242 showed G1 arrest/cell size decrease in trypanosomes, this was only found at lower drug concentrations in *L. major*, and at higher drug concentrations G2 arrest and increased cell size was observed instead. Thus, while the effects of specific inhibitors on trypanosomatids may resemble those seen against mammalian cells targeting specific TOR or PI3K targets, future studies will be required to more definitely establish the true mode(s) of action against the individual parasite species, which may differ.

It appears that the mTOR/PI3K inhibitors display generally superior activity against trypanosomatid growth over mTOR-selective inhibitors. This may be suggestive of the effect being mediated via inhibition of multiple trypanosomal PI3Ks, including PKKs such as TOR. With that in mind, efforts to identify the mechanism of action of these mTOR inhibitors in trypanosomatids will direct further medicinal chemistry efforts. Despite the lack of certainty of the mechanism of action of these compounds, the results we report in this work provide a validation for the repurposing approach as an efficient approach to identification of compounds that can potentially be effective in parasite killing.

### Conclusions

In summary, by application of the target repurposing approach, we have identified a series of established mTOR and mTOR/PI3K inhibitors that display a range of activity against the trypanosomatid parasites *T. brucei*, *T. cruzi*, and *Leishmania*. These compounds provide a promising starting point for discovery of new drugs for trypanosomal infections. While additional study is needed to determine the exact mechanism of action of these agents, these results indicate promising inroads to a new class of therapeutics. Encouragingly, the most potent and effective compound identified in these studies, NVP-BEZ235, is in clinical testing as an anticancer agent, and, if approved for this primary indication, may also warrant exploration as an anti-trypanosomal agent.
